# Perceived stress and anxiety as mediators linking self-disclosure to post-abortion depressive symptoms

**DOI:** 10.3389/fpsyt.2026.1766324

**Published:** 2026-03-05

**Authors:** Jin Xu, Xiulian Xu, Bingcui Sun, Ying Chen, Yan Huang, Xiaofang Cheng, Jing Zhao

**Affiliations:** 1 School of Nursing and Rehabilitation, Shandong University, Jinan, Shandong, China; 2 Department of Surgical Clinic, Qilu Hospital of Shandong University, Jinan, Shandong, China; 3 Nursing Theory and Practice Innovation Research Center, Shandong University, Jinan, Shandong, China; 4 Outpatient Department of Obstetrics and Gynecology, Qilu Hospital of Shandong University, Jinan, Shandong, China

**Keywords:** anxiety, depressive symptoms, distress disclosure, perceived stress, post-abortion women

## Abstract

**Objective:**

To examine the mediating roles of anxiety and perceived stress in the relationship between distress disclosure and depressive symptoms among women following induced abortion.

**Methods:**

A cross-sectional study was conducted with 359 post-abortion women recruited from a hospital in China. Participants completed self-report measures including the Distress Disclosure Index, Generalized Anxiety Disorder-7, Perceived Stress Scale-4, and Patient Health Questionnaire-9. Multiple mediation and subgroup analyses by marital status were performed using SEM model.

**Results:**

Distress disclosure had a significant total effect on depressive symptoms (β = -0.140, P < 0.001), but no direct effect (β = -0.017, P = 0.546). The association was fully mediated by anxiety and perceived stress. Subgroup analyses revealed that this mediating model was significant among unmarried women but not among married women.

**Conclusion:**

Distress disclosure is associated with depressive symptoms indirectly by reducing anxiety and perceived stress, particularly among unmarried women. These findings support integrated interventions combining emotional disclosure with anxiety and stress management to optimize psychological recovery for post-abortion women.

## 1 Introduction

Induced abortion is a common medical procedure worldwide, and its associated psychological health risks, particularly the high prevalence of post-abortion depression, have become a critical public health concern (1). As a core mechanism of emotional regulation and psychological adaptation, self-disclosure has long been recognized for its potential in alleviating psychological distress (2), a foundational finding that underpins modern research on its mental health benefits. Building on this classic work, contemporary studies (3–5) consistently link self-disclosure to reduced depressive symptoms, though the strength of this association varies by context and population. For example, distress disclosure on social media is specifically associated with depressive symptoms among young people (5), highlighting the role of disclosure strategies and contextual factors.

Nevertheless, the relationship between self-disclosure and depressive symptoms remains contentious. Cross-cultural comparative studies have revealed that the effect of self-disclosure on reducing depressive symptoms is significant among Taiwanese individuals with low mindfulness, but not among European Americans, indicating that its effectiveness is moderated by cultural background and individual traits (6). More critically, existing research has not systematically elucidated the mechanisms underlying this relationship, especially among women who have undergone induced abortion, a population facing unique psychosocial stressors (e.g., stigma, self-blame, future uncertainty) that differ from other groups.

Although previous research on assisted reproductive populations has examined the mediating effects of marital satisfaction and family decision-making power on the relationship between self-disclosure and depressive symptoms (7), the current study focuses instead on the mediating roles of perceived stress and anxiety symptoms, rather than interpersonal factors. This theoretical choice is based on the unique psychosocial challenges faced by women after abortion: their core psychological distress stems more from internal emotional impacts—such as fear of the procedure and uncertainty about the future—and cognitive threats, including stigma and self-blame, rather than directly from interpersonal relationship quality. Induced abortion can be regarded as a salient stressful event that triggers intense initial emotional reactions and negative cognitive appraisals. If individuals fail to effectively regulate these emotions through mechanisms such as self-disclosure, it may lead to persistent anxiety, heightened perceived stress, and ultimately, depressive symptoms.

Empirical evidence supports the mediating potential of perceived stress: self-disclosure helps individuals make sense of stressful experiences, reducing subjective stress burden and subsequent depression (8, 9). However, findings on anxiety as a mediator remain mixed—some studies show no significant effect (10), while others report anxiety reduction through self-disclosure (3, 11), warranting further investigation.

Notably, no previous research has systematically tested anxiety and perceived stress as dual mediators in depressive symptoms among post-abortion women. In summary, this study hypothesizes that self-disclosure alleviates depressive symptoms in women after induced abortion by reducing both perceived stress and anxiety.

## 2 Methods

### 2.1 Study design

#### 2.1.1 Participants and data collection

A cross-sectional study was conducted using convenience sampling. A total of 359 women who underwent induced abortion were recruited from the gynecology outpatient clinic of Qilu Hospital, Shandong University. After obtaining informed consent, research assistants administered all questionnaires electronically via Wenjuanxing. All items were set as mandatory to ensure complete data collection.

Inclusion criteria were: (1) age ≥ 18 years; (2) undergoing induced abortion; (3) literacy and ability to complete self-reported questionnaires; (4) clear consciousness and voluntary participation. Exclusion criteria included: (1) severe physical diseases (e.g., cardiovascular, respiratory, hepatic, renal, or malignant conditions); (2) current psychotropic medication use or structured psychotherapy (e.g., cognitive behavioral therapy); (3) cognitive impairment or communication difficulties; (4) incomplete survey responses.

#### 2.1.2 Sample size estimation

Sample size was estimated using G*Power 3.1. Based on a medium effect size (f² = 0.15) commonly observed in mediation studies, with α = 0.05 and power (1−β) = 0.95 for two mediators, the minimum required sample size was 269. The final sample of 359 participants provided adequate statistical power to detect significant effects.

### 2.2 Instruments

#### 2.2.1 Basic information

The basic information collected includes age (in years), whether living alone (yes/no), type of payment (medical insurance/self-payment), single parent status (yes/no), educational level (high school or below, associate degree, bachelor’s degree, master’s degree, doctoral degree), intimate relationship length (1 year or less, 1–3 years, 3–7 years, 7 years or more), smoking status (yes/no), and drinking status (yes/no).

#### 2.2.2 Patient health questionnaire

The PHQ-9 is a self-administered instrument used to assess the severity of depressive symptoms based on the DSM-IV diagnostic criteria (12). The Chinese version has been validated and demonstrates good reliability and validity (13). Each item is rated on a 4-point scale, and the total score is calculated by summing all items, with higher scores indicating more severe depressive symptoms.

#### 2.2.3 Generalized anxiety disorder-7

The GAD-7, developed by Spitzer et al., is a 7-item scale designed to measure the severity of anxiety symptoms (14). The Chinese version has also been psychometrically evaluated (15). Responses are given on a 4-point frequency scale, and the total score reflects the level of anxiety severity.

#### 2.2.4 Distress disclosure index

The Distress Disclosure Index (DDI), developed by Hessling and Kahn (16), is a widely used 12-item self-report instrument. Items are rated on a 5-point Likert-type scale, ranging from 1 (“strongly disagree”) to 5 (“strongly agree”). Reverse scoring is applied to items 2, 4, 5, 8, 9, and 10.

#### 2.2.5 Perceived stress scale-4

The PSS-4, developed by Cohen et al. (17), is a 4-item self-report scale measuring perceived stress over the past month. Items are rated on a 5-point frequency scale from *Never* to *Very often*. Questions 2 and 3 are reverse-scored.

### 2.3 Ethical considerations

This study was conducted in accordance with the Declaration of Helsinki and received ethical approval from the Research Ethics Committee of Qilu Hospital, Shandong University (Approval No: KYLL-202408-012-1). Written informed consent was obtained from all participants. All responses were kept strictly confidential, and no personally identifiable information was collected.

### 2.4 Statistical methods

Descriptive statistics were presented as means (standard deviations) and counts (percentages). A multiple mediation analysis was then conducted using R software (version 4.4.2), with both mediators (anxiety and perceived stress) tested simultaneously to examine the direct effect of self-disclosure on depressive symptoms and its indirect effects through the proposed mediators. To explore the potential moderating role of marital status, subgroup multiple mediation analyses were further performed separately for married and unmarried participants. The structural equation modeling (SEM) approach was adopted to fit the mediation model.

## 3 Results

**Table 1** shows the basic information of the participants. **Supplementary Table 1** presents the detailed baseline characteristics of the study participants stratified by marital status. The analysis included a total of 359 women who had undergone induced abortion. Among them, 214 (59.61%) were not married and 145 (40.39%) were married. Compared to unmarried participants, married individuals were significantly older and more likely to be living with others, use medical insurance, and have longer intimate relationships (all P < 0.001). Conversely, unmarried women reported significantly higher rates of smoking (P < 0.001) and drinking (P = 0.004). Perceived stress was significantly higher in the unmarried group (P < 0.001), while no significant differences were found in depressive symptoms (P = 0.540), anxiety symptoms (P = 0.667), or self-disclosure (P = 0.075).

**Table 1:** Characteristics of participants.

Characteristic	Total
N (%)	359 (100)
Age, years	27.29 ± 6.21
Living alone (%)	
No	266 (74.09)
Yes	93 (25.91)
Type of payment (%)	
Medical Insurance	130 (36.21)
Self-Payment	229 (63.79)
Single parent status (%)	
No	343 (95.54)
Yes	16 (4.46)
Educational level (%)	
High School or Below	39 (10.86)
Associate Degree	96 (26.74)
Bachelor’s Degree	188 (52.37)
Master’s Degree	32 (8.91)
Doctoral Degree	4 (1.11)
Intimate relationship length (%)	
1 Year or Less	100 (27.86)
1–3 Years	140 (39.00)
3–7 Years	46 (12.81)
7 Years or More	73 (20.33)
Smoking (%)	
No	333 (72.76)
Yes	26 (7.24)
Drinking (%)	
No	328 (91.36)
Yes	31 (8.64)
PHQ-9	6.13 ± 5.61
GAD-7	3.53 ± 5.18
PPS-4	6.55 ± 2.34
DDI-12	41.17 ± 7.93

**Figure 1:**
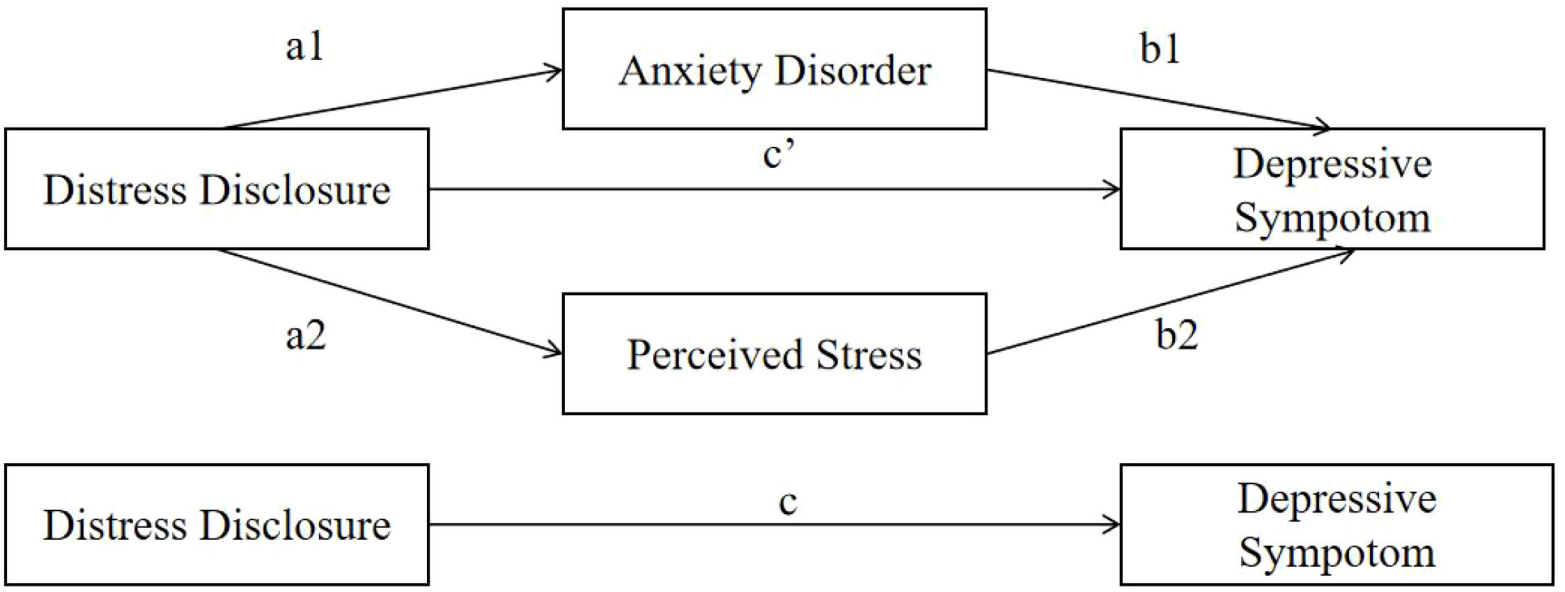
The mediating role of anxiety and perceived stress in the association between distress disclosure and depressive symptoms.

Main Mediation analysis results (**Table 2**; **Figure 1**) demonstrated a significant total effect of distress disclosure on depressive symptoms (β = -0.140, 95% CI [-0.210, -0.071], P < 0.001). Crucially, the direct effect of distress disclosure on depressive symptoms was not statistically significant (β = -0.017, 95% CI [-0.072, 0.038], P = 0.546), indicating complete mediation. The total indirect effect through both mediators was significant (β = -0.123, 95% CI [-0.178, -0.068], P < 0.001). Specifically, higher levels of distress disclosure significantly predicted lower anxiety (Path a1: β = -0.125, P < 0.001), which in turn predicted higher depressive symptoms (Path b1: β = 0.734, P < 0.001); the resulting indirect effect through anxiety was significant (β = -0.092, 95% CI [-0.142, -0.042]). Simultaneously, higher distress disclosure significantly predicted lower perceived stress (Path a2: β = -0.123, P < 0.001), and lower perceived stress predicted reduced depressive symptoms (Path b2: β = 0.256, P = 0.006); the indirect effect through this pathway was also significant (β = -0.031, 95% CI [-0.055, -0.008]). These results confirm that the relationship between distress disclosure and depressive symptoms is fully mediated by both anxiety symptoms and perceived stress.

**Table 2:** The mediating role of anxiety disorder and perceived stress in the association between distress disclosure and depressive symptoms (n = 359).

Effect path	Estimate	S.E.	CI. lower	CI. upper	P value
a1	-0.125	0.034	-0.192	-0.059	0.000
a2	-0.123	0.014	-0.151	-0.095	0.000
b1	0.734	0.039	0.657	0.811	0.000
b2	0.256	0.094	0.072	0.440	0.006
Total effect(c)	-0.140	0.036	-0.210	-0.071	0.000
Direct effect(c’)	-0.017	0.028	-0.072	0.038	0.546
Total indirect effect	-0.123	0.028	-0.178	-0.068	0.000
Indirect effect (Anxiety Disorder)	-0.092	0.025	-0.142	-0.042	0.000
Indirect effect (Perceived Stress)	-0.031	0.012	-0.055	-0.008	0.009

CI, confidence interval; Confidence intervals are calculated based on the bias-corrected bootstrap method; Mediators (anxiety disorder and perceived stress) were tested simultaneously.

Among unmarried women (n = 214; **Supplementary Table 2**), distress disclosure had a significant total effect on depressive symptoms (β = −0.176, 95% CI [−0.266, −0.085], P < 0.001) but no significant direct effect (β = −0.018, 95% CI [−0.094, 0.057], P = 0.635), and the total indirect effect was significant (β = −0.157, 95% CI [−0.229, −0.085], P < 0.001), indicating full mediation. In contrast, among married women (n = 145; **Supplementary Table 3**), neither the total effect (β = −0.094, 95% CI [−0.203, 0.015], P = 0.092) nor the total indirect effect of distress disclosure (β = −0.070, 95% CI [−0.157, 0.017], P = 0.117) was significant.

## 4 Discussions

This study provides empirical evidence clarifying the psychological mechanisms through which distress disclosure is associated with depressive symptoms in women following induced abortion. While a significant total effect was observed, the lack of a direct effect suggests that the relationship is fully mediated by both anxiety symptoms and perceived stress. These findings underscore the critical roles of emotional and cognitive processing in post-abortion mental health.

### 4.1 Total effect

The present study revealed a significant total effect of distress disclosure on depressive symptoms (β = –0.140, P < 0.001), which is highly consistent with existing literature (10). Higher levels of self-disclosure have been consistently associated with greater psychological well-being, more positive attitudes toward seeking professional help, and reduced depressive symptoms, particularly within psychotherapeutic contexts (18). These findings further support the potential of psychological interventions as preventive strategies in public health (2).

### 4.2 Direct effect

However, the direct effect of self-disclosure on depressive symptoms was not significant (β = –0.017, P = 0.546), indicating that its beneficial influence is not direct but fully mediated through reductions in both anxiety and perceived stress. This contrasts with previous studies involving assisted reproductive populations, which reported a partial mediation effect of self-disclosure on depression (19). One plausible explanation is that higher levels of disclosure do not uniformly predict lower depression due to individual differences and contextual factors. For instance, individuals with lower levels of anticipated discrimination and stigma-related stress are more inclined to disclose and experience better mental health outcomes after disclosure (20). Similarly, research among HIV-positive patients (21) found that neither disclosure rates nor recipients’ responses were significantly correlated with anxiety or depression; in some cases, high disclosure rates even increased distress due to stigmatizing reactions. Moreover, the emotional impact of disclosure depends heavily on contextual factors such as timing and social support availability (22). Repeated emotional disclosure may also trigger negative responses from partners (e.g., emotional invalidation), linked to relational distancing and potentially exacerbating depressive symptoms (23).

Additionally, prior research has shown that 22.5% of Chinese women who underwent abortion experienced depressive symptoms, with high perceived stress and low social support being major risk factors (7). In light of our findings, it can be inferred that within the specific context of induced abortion, characterized by high stress, stigma, and emotional conflict, self-disclosure alone may be insufficient to directly be linked to depression. Instead, all its benefits are likely mediated through the reduction of acute stress responses and anxiety symptoms.

### 4.3 Psychological mechanisms

The mediating effects occur through two distinct pathways: perceived stress and anxiety symptoms. On one hand, self-disclosure facilitates cognitive reappraisal, thereby reducing perceived stress. The process of articulating distressing experiences enables women to actively reconstruct and integrate their emotions, leading to improved emotional awareness and more rational understanding of the abortion experience (24). This reduces self-blame and internalized stigma (25). Repeated verbalization also promotes cognitive desensitization, gradually diminishing emotional arousal related to the event and effectively alleviating perceived stress (26). Thus, through cognitive restructuring, self-disclosure alters an individual’s appraisal and tolerance of stress, ultimately mitigating depressive symptoms.

On the other hand, self-disclosure relieves anxiety through emotional ventilation. Authentic expression of emotions such as pain, fear, and sorrow helps release pent-up emotional energy and disrupts inhibitory processes (27). This not only provides an internal channel for emotional release but also reduces feelings of isolation through external empathy and social support, thereby directly alleviating anxiety. From a neuroendocrine perspective, emotional ventilation can attenuate hyperactivity of the hypothalamic-pituitary-adrenal (HPA) axis and reduce the secretion of stress hormones, establishing a physiological basis for anxiety reduction. This mechanism is particularly critical among stigmatized populations (26), further explaining the anxiety-mediated pathway identified in this study.

It is important to emphasize that this study distinguishes between two independent mediators: “perceived stress”, which representing cognitive appraisal and “anxiety symptoms”, which reflecting emotional experience. While previous studies may have focused on only one of these mechanisms, our results demonstrate that both pathways independently mediate the relationship between self-disclosure and depression. This indicates that self-disclosure influences depressive symptoms both by modifying cognitive evaluation of stress and by alleviating emotional anxiety, thereby enhancing our understanding of the underlying mechanisms and highlighting the value of multi-dimensional psychological interventions.

### 4.4 Cultural effect of marital status

Notably, marital status may act as a significant cultural moderator in the present dual-mediation model, which is highly embedded in the Chinese sociocultural context. Married women in China usually receive more stable family support and higher social recognition (28), which can buffer the stigmatization pressure after distress disclosure and weaken the mediating role of perceived stress In contrast, unmarried women face stronger social stigma, higher levels of self-blame, and less available social support regarding induced abortion in traditional Chinese culture (29); consequently, the mediating effect of perceived stress tends to be stronger in this subgroup. Such moderating patterns further enrich our culturally adapted framework by highlighting how marital status shapes cognitive and emotional pathways in post-abortion psychological recovery.This study advances understanding by demonstrating full mediation through anxiety and stress, emphasizing the need for culturally sensitive, multi-dimensional interventions.

## 5 Strengths and implications

The findings offer important practical implications for designing psychological interventions for post-abortion women. Intervention should integrate disclosure promotion with structured stress management and anxiety reduction techniques. Rather than merely encouraging self-disclosure, interventions should specifically target the mediating variables—anxiety and perceived stress. For example, to enhance the clinical practicability of the intervention for students enrolled in the Master of Mental Health Nursing program, this article further specifies detailed strategies for integrating mindfulness training and relaxation training into the self-disclosure intervention process: In one-on-one psychological counseling sessions for post-abortion women (typically lasting 20–30 minutes), mental health nurses first guide women to disclose their distressing thoughts and emotions (e.g., fear, self-blame) in a safe, non-judgmental environment. Upon completion of disclosure, nurses immediately lead them through 5–8 minutes of structured mindfulness exercises (e.g., focused breathing meditation, body scan) to help individuals ground themselves in the present moment and reduce acute anxiety arousal. Subsequently, 3–5 minutes of progressive muscle relaxation training is conducted, guiding individuals to systematically tense and relax each muscle group to alleviate physical tension associated with perceived stress. Meanwhile, nurses establish a connection between disclosure and the relief of stress and anxiety through verbal guidance—for instance, explaining to the client, “It is very brave of you to share these feelings; this set of relaxation exercises will help your body and mind release the stress you are bearing”—thereby strengthening the link between disclosure behavior and symptom relief. This integrated intervention protocol is feasible in hospital settings, as it requires simple equipment, aligns with the routine nursing process for post-abortion care, and can be flexibly adjusted according to the individual needs of patients.

From a cross-cultural perspective, this study provides valuable empirical evidence for psychological rehabilitation after abortion in the Chinese context. Unlike Western intervention models that emphasize individual expression and emotional release, Chinese women may place greater emphasis on social support availability, family acceptance, and stigma avoidance during self-disclosure (30). Therefore, the anxiety- and stress-mediated pathway identified in this study not only deepens the theoretical understanding of self-disclosure but also offers a culturally adaptive framework for developing targeted interventions in diverse cultural settings.

## 6 Limitations and future directions

Several limitations of this study should be acknowledged. First, the cross-sectional design, with all variables measured at a single time point, precludes causal inference. Caution is warranted when interpreting the causal pathways among self-disclosure, anxiety, stress, and depressive symptoms, due to potential reverse causality or unmeasured confounding factors. Second, the sample was recruited from a single medical institution, which may limit the generalizability of the findings. Third, self-report measures may inflate associations; need to be replicated in diverse cultural contexts. Fourth, regarding cultural specificity, this study was conducted exclusively in a Chinese context, where abortion-related stigma, family dynamics, and attitudes toward emotional expression differ from Western or other non-Chinese cultures. What’s more, this study employed a convenience sampling method to recruit participants, which constitutes a key methodological limitation. Convenience sampling may introduce selection bias, as the sample is likely to overrepresent individuals who are more willing to participate.

To address these limitations, future research should adopt longitudinal designs with repeated measurements at multiple time points post-abortion to clarify temporal dynamics and strengthen causal evidence for the proposed mediation pathways. Furthermore, incorporating additional factors—such as recipients’ responses, level of social support, self-compassion, and cultural variables—could help develop more comprehensive and nuanced mediating or moderating models, elucidating the boundary conditions and psychological mechanisms through which self-disclosure operates. Future studies should include more diverse cultural samples and adopt probability sampling strategies (e.g., stratified random sampling) to improve external validity.

## 7 Conclusion

In conclusion, this study demonstrates that distress disclosure exerts its beneficial effect on depressive symptoms among post-abortion women indirectly, through the mediating pathways of reduced anxiety and lower perceived stress. This study advances understanding by demonstrating full mediation through anxiety and stress, emphasizing the need for culturally sensitive, multi-dimensional interventions. This research contributes to a more culturally and contextually nuanced understanding of post-abortion psychological recovery and offers practical insights for developing targeted support frameworks for this vulnerable population.

## Data Availability

The raw data supporting the conclusions of this article will be made available by the authors, without undue reservation.
