# Supplementary material for: Perceived stress and anxiety as mediators linking self-disclosure to post-abortion depressive symptoms
Source: Front Psychiatry. 2026 Mar 5;17:1766324. doi: 10.3389/fpsyt.2026.1766324 (PMC12999907; doi:10.3389/fpsyt.2026.1766324)
Supplement: Supplementary file 1 [file Table1.docx]

**Supplementary table 1**. Characteristics of participants by Marital Status.

| Characteristic | Not Married | Married | P value |
| --- | --- | --- | --- |
| N(%) | 214(59.61) | 145(40.39) |  |
| **Age, years** | 23.64±3.14 | 32.69±5.66 | 0.000 |
| Living alone(%) |  |  | 0.000 |
| No | 126(58.88) | 140(96.55) |  |
| Yes | 88(41.12) | 5(3.45) |  |
| Type of Payment(%) |  |  | 0.000 |
| Medical Insurance | 44(20.56) | 86(59.31) |  |
| Self-Payment | 170(79.44) | 59(40.69) |  |
| Single Parent Status(%) |  |  | 0.446 |
| No | 203(94.84) | 140(96.55) |  |
| Yes | 11(5.14) | 5(3.45) |  |
| Educational level(%) |  |  | 0.077 |
| High School or Below | 25(11.68) | 14(9.66) |  |
| Associate Degree | 63(29.44) | 33(22.76) |  |
| Bachelor's Degree | 112(52.34) | 76(52.41) |  |
| Master's Degree | 12(5.61) | 20(13.79) |  |
| Doctoral Degree | 2(0.93) | 2(1.38) |  |
| Intimate Relationship Length(%) |  |  | 0.000 |
| 1 Year or Less | 88(41.12) | 12(8.28) |  |
| 1-3 Years | 107(50.00) | 33(22.76) |  |
| 3-7 Years | 14(6.54) | 32(22.07) |  |
| 7 Years or More | 5(2.34) | 68(49.60) |  |
| Smoking(%) |  |  | 0.000 |
| No | 189(88.32) | 144(99.31) |  |
| Yes | 25(11.68) | 1(0.69) |  |
| Drinking(%) |  |  | 0.004 |
| No | 188(87.85) | 140(96.55) |  |
| Yes | 26(12.15) | 5(3.45) |  |
| **PHQ-9** | 6.28±5.51 | 5.91±5.77 | 0.540 |
| **GAD-7** | 3.63±5.27 | 3.39±5.07 | 0.667 |
| **PPS-4** | 6.92±2.13 | 6.01±2.53 | 0.000 |
| DDI-12 | 41.06±7.13 | 41.34±8.24 | 0.075 |

**Supplementary table 2**. The mediating role of anxiety disorder and perceived stress in the association between distress disclosure and depressive symptoms in unmarried women (n = 214).

| Effect path | Estimate | S.E. | CI. lower | CI. upper | P value |
| --- | --- | --- | --- | --- | --- |
| a1 | -0.186 | 0.045 | -0.274 | -0.098 | 0.000 |
| a2 | -0.120 | 0.017 | -0.153 | -0.086 | 0.000 |
| b1 | 0.698 | 0.051 | 0.597 | 0.799 | 0.000 |
| b2 | 0.231 | 0.136 | -0.035 | 0.496 | 0.089 |
| Total effect(c) | -0.176 | 0.046 | -0.266 | -0.085 | 0.000 |
| Direct effect(c’ ) | -0.018 | 0.039 | -0.094 | 0.057 | 0.635 |
| Total indirect effect | -0.157 | 0.037 | -0.229 | -0.085 | 0.000 |
| Indirect effect (Anxiety Disorder) | -0.130 | 0.033 | -0.194 | -0.066 | 0.000 |
| Indirect effect (Perceived Stress) | -0.028 | 0.017 | -0.060 | -0.005 | 0.098 |

Note: CI: confidence interval; Confidence intervals are calculated based on the bias-corrected bootstrap method; Mediators (anxiety disorder and perceived stress) were tested simultaneously.

**Supplementary table 3**. The mediating role of anxiety disorder and perceived stress in the association between distress disclosure and depressive symptoms in married women (n = 145).

| Effect path | Estimate | S.E. | CI. lower | CI. upper | P value |
| --- | --- | --- | --- | --- | --- |
| a1 | -0.046 | 0.051 | -0.146 | 0.054 | 0.364 |
| a2 | -0.125 | 0.023 | -0.171 | -0.079 | 0.000 |
| b1 | 0.796 | 0.062 | 0.675 | 0.917 | 0.000 |
| b2 | 0.263 | 0.135 | -0.001 | 0.527 | 0.051 |
| Total effect(c) | -0.094 | 0.056 | -0.203 | 0.015 | 0.092 |
| Direct effect(c’ ) | -0.024 | 0.041 | -0.106 | 0.057 | 0.557 |
| Total indirect effect | -0.070 | 0.044 | -0.157 | 0.017 | 0.117 |
| Indirect effect (Anxiety Disorder) | -0.037 | 0.041 | -0.116 | 0.043 | 0.365 |
| Indirect effect (Perceived Stress) | -0.033 | 0.018 | -0.068 | 0.002 | 0.066 |

Note: CI: confidence interval; Confidence intervals are calculated based on the bias-corrected bootstrap method; Mediators (anxiety disorder and perceived stress) were tested simultaneously.
